# Generationenübergreifende Betreuung von Patienten mit tachykarden Rhythmusstörungen

**DOI:** 10.1007/s00112-022-01591-9

**Published:** 2022-09-09

**Authors:** Manfred Marx, Matthias Gass, Ina Michel-Behnke

**Affiliations:** 1 Klinische Abteilung für Pädiatrische Kardiologie, Universitätsklinik für Kinder- und Jugendheilkunde, Medizinische Universität Wien, Währinger Gürtel 18–20, 1090 Wien, Österreich; 2 Pädiatrische Kardiologie, Universitäts-Kinderklinik Zürich, Zürich, Schweiz

**Keywords:** Ionenkanäle, Antiarrhythmika, Katheterablation, Genetische Arrhythmiesyndrome, Kardiale Elektrophysiologie, Ion channels, Antiarrhythmic drugs, Catheter ablation, Inherited arrhythmogenic diseases, Cardiac electrophysiology

## Abstract

Die Versorgung von Patienten mit Rhythmusstörungen hat sich in den letzten Jahrzehnten von einer rein konservativ medikamentösen Therapie zu einer echten kurativen Therapie mit Beseitigung des arryhthmogenen Substrats durch technisch immer ausgereiftere Möglichkeiten im Sinn der elektrophysiologische Untersuchung (EPU) und Ablation entwickelt. Parallel dazu haben sich in pädiatrisch-kardiologischen Zentren rhythmologische Spezialambulanzen zur Betreuung von Patienten mit Ionenkanalerkrankungen etabliert. Deren Aufgabe besteht in der generationenübergreifenden Betreuung von ganzen Familien, mit dem Ziel, präventiv, durch entsprechende Beratung und Führung, maligne Rhythmusstörungen primär zu verhindern.

## Abstract (en)

Within the last decades the treatment options for patients with arrhythmias have developed from a purely conservative drug treatment to a really curative treatment with removal of the arrhythmogenic substrate by continuously more sophisticated possibilities in the sense of electrophysiological techniques and ablation. Parallel to this, special outpatient departments of cardiac rhythmology for the care of patients with ion channelopathies have also become established in pediatric cardiology centers. Their task is the intergenerational care of whole families, with the aim of primarily preventing malignant arrhythmias by appropriate counselling and guidance.

## Lernziele

Nach der Lektüre dieses Beitrags …

- sind Sie für die Patientengruppe sensibilisiert, die eine weiterführende Diagnostik/Betreuung benötigt.
- haben Sie einen Überblick über den aktuellen Stand der interventionellen Therapie von Rhythmusstörungen.
- können Sie die wichtigsten genetischen Erkrankungen in einer Rhythmusambulanz für Ihren Alltag einordnen.
- verstehen Sie die Notwendigkeit einer generationenübergreifenden Familienbetreuung zur Prävention von malignen Rhythmusstörungen.

## Einleitung

Bei der ambulanten und interventionellen Betreuung von Patienten mit tachykarden Rhythmusstörungen haben sich in den letzten Jahrzehnten 3 wesentliche therapeutische Ansätze entwickelt. Der erste besteht aus einer konservativen, medikamentösen Therapie, die v. a. Säuglingen und Kleinkindern vorbehalten bleibt und die Zeit bis zum zweiten echten kurativen Therapieansatz mit Beseitigung des arryhthmogenen Substrats im Sinne der elektrophysiologischen Untersuchung (EPU) und Ablation überbrücken soll.

Parallel dazu haben sich in pädiatrisch-kardiologischen Zentren in zunehmendem Ausmaß rhythmologische Spezialambulanzen zur Betreuung von Patienten mit „genetischen Arrhythmiesyndromen“ (GAS) etabliert. Im Folgenden werden die Erfahrungen bezüglich dieser 3 Therapieansätze wiedergegeben.

## Therapeutische Ansätze

### Medikamentöse Therapie

**Figure 1:**
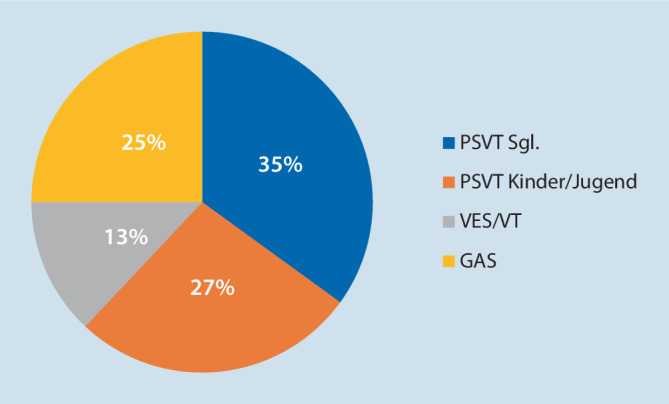

Wie in Abb. 1 dargestellt, sind 35 % der Patienten in der Rhythmusambulanz der Universitätsklinik für Kinder- und Jugendheilkunde, Medizinische Universität Wien, Neugeborene und Säuglinge mit paroxysmalen supraventrikulären Tachykardien (PSVT).

Die häufigste Ursache einer fetalen, beim Neugeborenen und im Säuglingsalter auftretenden PSVT ist eine zusätzliche akzessorische Leitungsbahn als elektroanatomische Grundlage für AV-Reentry-Tachykardien (AVRT). Fokale atriale Tachykardien (FAT) sind bei herzgesunden Kindern nur zu einem geringen Prozentsatz von < 5 % anzutreffen. Fokale Tachykardien aus dem AV-Knoten, auch junktionale ektope Tachykardien (JET) genannt, sind in der ambulanten Versorgung extrem selten, postoperativ allerdings nach Operationen an der Herz-Lungen-Maschine ein häufigeres, jedoch zumeist passageres Problem. In seltenen Fällen können diese zum schwersten „low cardiac output“ führen. Ebenfalls selten sind Vorhofflattern oder intraatriale Reentrytachykardien (IART), wobei Letztere bei Zustand nach Herzoperationen im Vorhofbereich möglich sind.

Bereits die Versorgung von Patienten mit einer fetalen Tachykardie stellt eine generationenübergreifende Maßnahme dar und sollte unbedingt im Team festgelegt werden, da eine Fortsetzung der Therapie beim Neugeborenen sehr wahrscheinlich ist.

Zur Therapie von fetalen Arrhythmien werden sehr unterschiedliche Strategien verfolgt. In der Rhythmusambulanz der Universitätsklinik für Kinder- und Jugendheilkunde, Medizinische Universität Wien, werden bei Vorhofflattern Sotalol und bei supraventrikulären Tachykardien (SVT) Flecainid als First-Line-Therapie, evtl. in Kombination mit Digoxin, verabreicht. Wegen des schlechten diaplazentaren Wirkstoffübertritts kann es bei der Gabe von Amiodaron manchmal schwierig sein, einen ausreichenden Spiegel beim Fetus zu erreichen.

Eine Übersicht der verwendeten Dosierungen zur transplazentaren Therapie findet sich in Tab. 1.

**Table 1:** 

Wirkstoff	Dosierung
Digoxin	LD 1,5–2 mg/Tag p.o. über 2 Tage ED 0,25–0,75(–1) mg/Tag p.o. Ziel: Digoxinspiegel: 2–2,5(–3)ng/ml
Sotalol	160–320(–480) mg/Tag p.o. in 2–3 ED
Flecainid	(100–)300 mg/Tag p.o. in 3 ED
Amiodaron	LD 1600–2000 mg/Tag p.o. für 2 Tage LD bei Medikamentenumstellung: 800–1200 mg/Tag p.o. für 2 Tage ED 200–600 mg/Tag p.o.

*LD* „loading dose“, *ED* Erhaltungsdosis

^a^Die Angaben erfolgen ohne Gewähr

Allen medikamentösen Therapieansätzen gemeinsam ist das Ziel der größtmöglichen Sicherheit

Bei Neugeborenen und Säuglingen mit einem Wolff-Parkinson-White(WPW)-Syndrom (overte Präexzitation und bereits stattgefundene Tachykardie) oder der AVRT bei einer ausschließlich retrograd leitenden Bahn („concealed pathway“) steht die Prävention eines Rezidivs der SVT im Vordergrund. Da es im 1. Lebensjahr durch Apoptose der akzessorischen Fasern häufig zum spontanen Sistieren der Tachykardien kommt, stellt die oft nur vorübergehende medikamentöse Therapie ein sinnvolles Konzept dar. Zu den verfügbaren Optionen gehören die Klasse-1C-Antiarrhythmika Propafenon oder Flecainid, die Gruppe der β‑Blocker wie z. B. Propranolol oder andere mehr kardioselektive β‑Blocker (Metoprolol, Atenolol) sowie die Klasse-III-Medikamente Sotalol und Amiodaron. Digoxin hat kaum mehr einen Stellenwert. Da Digoxin jedoch die antegrade effektive Refraktärperiode einer akzessorischen Leitungsbahn verkürzen kann und eine hochfrequente atrioventrikuläre Überleitung während Vorhofflattern und -flimmern begünstigt, ist sein Gebrauch bei Patienten mit einem Präexzitationssyndrom in jeder Altersstufe kontraindiziert [2]. Verapamil wurde mit irreversibler Hypotonie und Asystolie bei Säuglingen und Kleinkindern in Verbindung gebracht und sollte v. a. im 1. Lebensjahr nicht verabreicht werden. Allen medikamentösen Therapieansätzen gemeinsam ist das Ziel der größtmöglichen Sicherheit. Auch wenn in der Literatur keine Priorisierung eines bestimmten Antiarrhythmikums erkennbar ist, ist der beste „approach“ immer derjenige, mit dem individuell und institutionell die meiste Erfahrung vorliegt. Der Ansatz der Autoren des vorliegenden Beitrags ist, zu Beginn eher aggressiv zu therapieren, im Sinne einer Kombination von Klasse-1C-Medikamenten und β‑Blockern – alternativ Amiodaron, wobei Flecainid und Amiodaron mit etwa 80 % fast dieselben Erfolgsraten aufweisen [3]. Diese Therapie wird innerhalb der nächsten 3 Monate, wenn möglich, deeskaliert. Als kardiale Nebenwirkungen treten unter der Gabe von Flecainid (oder Propafenon) eher QRS-Verbreiterungen auf; bei Amiodaron stehen die Bradykardien und QTc-Verlängerungen im Vordergrund [3]. Eine Konzentrationserhöhung des thyreoidstimulierenden Hormons (TSH) – und der Beginn einer latenten oder manifesten Hypothyreose, die deutlich häufiger auftritt als die Hyperthyreose – kommt bei bis zu 50 % der mit Amiodaron behandelten Patienten vor, wobei Neugeborene und junge Säuglinge besonders stark davon betroffen sind [4]. Diese Auslenkungen können bereits sehr früh auftreten. Ein möglicher Zeitplan für Kontrolluntersuchungen ist in Tab. 2 dargestellt.

**Table 2:** 

Zeitpunkt	Schilddrüsenhormone	Weitere Untersuchungen/Untersuchungsparameter
Vor Beginn (wenn möglich)	TSH, fT_3_	–
2 Wochen	TSH, fT_3_	Transaminasen
4 Wochen	TSH, fT_3_	Augenuntersuchung, Transaminasen
8 Wochen	TSH, fT_3_	–
Danach alle 2 bis 3 Monate	TSH, fT_3_	Transaminasen
3 Monate nach Absetzen des Amiodarons	TSH, fT_3_	Augenuntersuchung

*fT*_*3*_ freies Trijodthyronin, *TSH* thyreoidstimulierendes Hormon

Für eine Übersicht der Akut- und Dauertherapie wird auf andere Literaturstellen verwiesen [5, 6].

### Interventionelle Therapie (elektrophysiologische Untersuchung und Ablation)

Bei Kindern mit einem Körpergewicht über 15 kg und Jugendlichen mit häufig auftretenden, symptomatischen Tachykardien ist der Ablationstherapie als kausale Behandlungsmaßnahme aufgrund der hohen Sicherheit und Effizienz der Vorzug zu gegeben [7]. Die Erfolgsraten betragen > 95 % in Abhängigkeit von der Lokalisation der Leitungsbahn; das AV-Block-Risiko beträgt < 1 % [7].

Patienten mit intermittierender Präexzitation oder symptomfreie Patienten mit overter Präexzitation (im EKG immer eine Delta-Welle vorhanden) stellen weiterhin eine klinische Herausforderung dar. Allerdings findet sich auch bei Patienten mit intermittierender Präexzitation dieselbe Anzahl von Bahnen mit malignen elektrophysiologischen Leitungskapazitäten wie bei Patienten mit permanenter Präexzitation; auch das Risiko einer „Fast-broad-irregular“(FBI)-Tachykardie bei rascher Überleitung von Vorhofflimmern über die akzessorische Bahn ist vergleichbar [8]. Das Verschwinden einer overten Präexzitation während der Ergometrie ist nicht immer einfach zu erkennen, da die gute antegrade Leitfähigkeit des AV-Knotens bei Jugendlichen z. B. die Präexzitation eines Bypass-Trakts (BPT) maskieren kann. Zusätzlich erschweren Bewegungsartefakte die Interpretation, sodass als prognostisches Kriterium nur der plötzliche und vollständige Verlust der Präexzitation gewertet werden darf, um daraus auf eine lange antegrade effektive Refraktärperiode (ERP) zu schließen [9]. Während die Ablation bei symptomatischen Patienten die Methode der Wahl darstellt, kann sie auch bei symptomfreien Kindern und Jugendlichen ab dem 5. Lebensjahr erwogen werden und ist hier eine Klasse-IIB-Indikation [10]. Da etwa 1 % der Todesfälle bei Sportlern auf ein WPW-Syndrom zurückzuführen sind [11], gelten für Wettkampf-, aber auch für engagierte Freizeitsportler besonders strenge Kriterien; in Europa wird bei WPW-Patienten eine EPU und evtl. eine Ablation gefordert [12]. Für Kinder und Jugendliche gibt es keine detaillierten Richtlinien, hier muss v. a. in der Altersgruppe zwischen 5 und 10 Jahren sehr individuell in Abhängigkeit von der Intensität der körperlichen Belastung, der Lokalisation des BPT und dem Risiko der Ablation entschieden werden [13]. Kinder über 10 Jahre sollten eine Sporterlaubnis (sowohl für Schul- als auch Wettbewerbssport) nur nach erfolgter EPU und (wenn möglich) Ablation erhalten.

**Figure 2:**
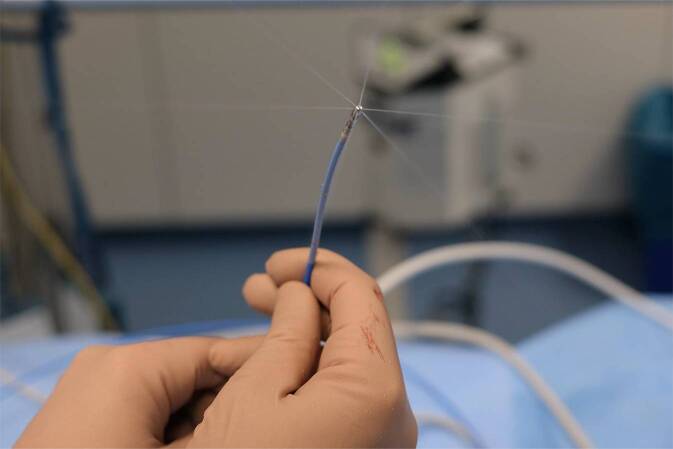

Moderne 3D-elektroanatomischer Mapping- und Navigationssysteme zur Erstellung von Aktivation‑, Substrat- und Stimulation-Maps haben die Katheterablation unter Verwendung von gekühlten Ablationskathetern (Abb. 2) zu einer Höchstpräzisionsmethode gewandelt. Die Möglichkeit der transseptalen Punktion schon im Kindesalter erleichtert den Zugang zu linksatrialen Substraten. Für ausführliche technische Details wird auf die Literatur verwiesen [14].

**Figure 3:**
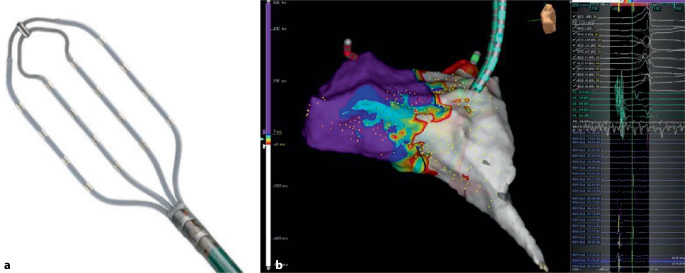

Mit aktuellen Features wie „open windows mapping“ können akzessorische Bahnen auch visualisiert werden. Wie in Abb. 3 dargestellt, hilft das Open window mapping unter Verwendung eines hochauflösenden, multipolaren Katheters (HD-Grid®, Abbott, Chicago, IL, USA), akzessorische Bahnen räumlich darzustellen und die elektrische Erregungsausbreitung über die Bahn zu visualisieren.

## Genetische Arrhythmiesyndrome

Den GAS liegt eine meistens autosomal-dominant vererbte Genmutation zugrunde; diese beeinflusst die Funktion der Ionenkanäle der Kardiomyozyten. Arrhythmiesyndrome mit struktureller Veränderung finden sich bei der arrhythmogenen rechtsventrikulären Kardiomyopathie (ARVC) und bei Kardiomyopathien aufgrund einer Laminopathie (Defekte der Zellkernhülle). In einer Autopsieserie von an ARVC verstorbenen Patienten waren 13 % jünger als 18 Jahre [15]. Bei den sog. Ionenkanalerkrankungen (Long-QT-Syndrom [LQTS], Short-QT-Syndrom, Brugada-Syndrom [BrS] und katecholaminerge polymorphe ventrikuläre Tachykardie [CPVT]) finden sich keine zusätzlichen strukturellen Veränderungen. Die letztgenannten Erkrankungen, auch als primär elektrische Erkrankungen bekannt, sind für etwa 30 % aller Fälle des plötzlichen kardialen Todes („sudden cardiac death“, SCD) bei Kindern und Jugendlichen verantwortlich [16]. Die beschriebenen Zahlen verdeutlichen die dringliche Notwendigkeit einer frühzeitigen Identifizierung der Patienten, die über mögliche Screeningprogramme oder über ein multidisziplinäres, generationenübergreifendes Modell in Zusammenarbeit mit Allgemeinmedizinern, Internisten und Genetikern unter gemeinsamer Nutzung eines „Registers“ erreicht werden kann.

Obwohl – wie z. B. beim LQTS – die Prävalenz der Erkrankung durch ein EKG-Screening mit nachfolgender genetischer Analyse von Verdachtsfällen sehr gut bestimmt wurde [17], haben sich Screeningprogramme in den meisten Ländern nicht durchsetzen können. Die Gründe sind gerade beim LQTS die möglicherweise mangelnde Reproduzierbarkeit, Spezifität und Sensitivität des EKG [18], die Angst vor Über- oder Nichtdiagnose und nicht zuletzt die Schwierigkeit im klinischen Alltag, eine einmal erstellte Verdachtsdiagnose endgültig zu exkludieren.

Primär elektrische Erkrankungen verursachen ca. 30 % der SCD-Fälle im Kindes- und Jugendalter

Dem von den Autoren favorisierten Ansatz des multidisziplinären, generationenübergreifenden Modells in Zusammenarbeit mit Allgemeinmedizinern, Internisten und Genetikern unter gemeinsamer Nutzung eines „Registers“ steht häufig der Datenschutz, der die direkte Einbindung aller Berufsgruppen und aller Familienmitglieder nach einer positiven genetischen Untersuchung zurzeit noch verhindert, im Weg.

Wesentliche Aufgaben einer modernen Rhythmusambulanz sind das Akquirieren/Erkennen und Betreuen von Patienten mit einer GAS zur Verhinderung des SCD. Im Weiteren folgt daher ein Überblick der genannten Erkrankungen aus klinischer Sicht.

### Arrhythmogene rechtsventrikuläre Kardiomyopathie

Die ARVC stellt ein möglicherweise unterdiagnostiziertes Krankheitsbild im Kindes- und Jugendalter dar. Die Prävalenz beträgt 1:5000 bzw. in einer Untersuchung sogar 1:1000 [19] und ist deutlich höher als bei den meisten angeborenen Stoffwechselerkrankungen, auf die heute gescreent wird. Zur Diagnosestellung gelten dieselben Kriterien wie bei Erwachsenen [20, 21]. Repolarisationsveränderungen sind ein sehr früher und sensitiver phänotypischer Marker der Erkrankung, ausreichend spezifisch wird dieses Zeichen jedoch erst jenseits des 14. Lebensjahres, da bei älteren gesunden Teenagern rechtspräkordiale T‑Negativierungen nur mehr in 4 % der weiblichen und in 1 % aller männlichen Probanden zu finden sind [22]. Die Depolarisationsverzögerung (Epsilon-Welle) als weiteres sensitives diagnostisches Kriterium kann im Kindes- und Jugendalter durch den relativ häufig vorkommenden inkompletten Rechtsschenkelblock „maskiert“ werden. Hilfreich ist vielleicht die Tatsache, dass auch bei einem kompletten Rechtsschenkelblock T‑Negativierungen in V_1_–V_4_ eher selten vorkommen, für die ARVC jedoch typisch wären.

Benigne ventrikuläre Extrasystolen (VES) bzw. die benigne Rechtsventrikuläre-Ausflusstakt-Tachykardie (RVOT-VT) und eine ventrikuläre Tachykardie (VT) bei ARVC haben beide eine linksschenkelblockartige Morphe. Es gilt daher bei jedem Patienten mit VES aus dem RV/RVOT, eine ARVC auszuschließen.

Die Prävalenz der ARVC ist deutlich höher als die der meisten angeborenen Stoffwechselerkrankungen

Während bei Erwachsenen das Kardio-MRT als Goldstandard zum Nachweis der typischen Veränderungen gilt, trifft dies für das Kindesalter nur bedingt zu [23]. Ausgeprägte Myokardverdünnungen, Fibrose, Aneurysmen der rechtsventrikulären (RV‑)Wand und Fettgewebseinlagerungen stellen zwar sehr typische, aber auch eher spät auftretende Veränderungen der Erkrankung dar, wobei die arrhythmogenen Ereignisse zumeist schon früher einsetzen. *Cave*: Ein normaler MRT-Befund, dem typischen Erwachsenenprotokoll entsprechend, schließt eine ARVC nicht sicher aus. Die Interpretation der Untersuchung ist höchst untersucherabhängig und sollte nur wenigen spezialisierten Zentren vorbehalten bleiben. Aus den genannten Gründen ist das MRT auch zum Screening von Nachkommen nur sehr eingeschränkt hilfreich [24].

Wie in Tab. 3 dargestellt, sollte bei einer ARVC, soweit wie möglich, auf jegliche sportliche Aktivität verzichtet werden.

**Table 3:** 

Risiko	ARVC-Typ	Freizeitaktivität	Schulturnen	Wettkampfsport	Bemerkungen
IR	Genetisch pos./phänotyp. neg.	+/−	+/−	−	–
HR	Genetisch pos./phänotyp. pos.	−	−	−	Evtl. ICD

*Beachte*: Sportempfehlungen sind immer Einzelfallentscheidungen nach detaillierter Aufklärung der Patienten

*ICD* implantierbarer Kardioverter-Defibrillator*, IR* „intermediate risk“, *HR* „higher risk“; *+* erlaubt, *–* nicht erlaubt

### Laminopathien

Obwohl Patienten mit Laminopathien (LMNA) in einer pädiatrischen Rhythmusambulanz insgesamt selten sind, sollte bei familiärer Häufung von dilatativen Kardiomyopathien (DCMP), v. a. wenn auch Reizleitungsstörungen vorliegen, an eine LMNA gedacht werden. Dysrhythmien (supraventrikuläre Extrasystolen [SVES], atriale Arrhythmien) und Überleitungsstörungen mit der Notwendigkeit einer Herzschrittmacherimplantation (3 % der Patienten im Alter zwischen 10 und 20 Jahren) können schon frühzeitig auftreten [25]. Ebenso sind VES häufig und haben zumeist eine schlechte Prognose [26]. Bei Patienten mit VES vom „uncommon type“ (ungewöhnlicher Lagetyp, Zunahme der VES unter Belastung) und „muskulären Symptomen“ muss eine LMNA ausgeschlossen werden. Eine Konzentrationserhöhung der Kreatinkinase (CK) kommt nur in 30 % der Fälle vor [27]. Die hohe Penetranz der kardialen Symptome nimmt mit dem Alter kontinuierlich zu, 60-Jährige sind zu 100 % symptomatisch [25]. Die LMNA ist eine typische Erkrankung, bei der weitere Betroffene der nächsten Generationen v. a. durch die Zusammenarbeit mit Kollegen aus dem Erwachsenenbereich erkannt werden können und wegen der schlechten Prognose auch erkannt werden müssen. Alle Patienten mit LMNA sollten an Tertiärzentren betreut werden, da die Möglichkeit einer der Versorgung mit einem implantierbaren Kardioverter-Defibrillator (ICD) oder einer Herztransplantation frühzeitig in Betracht gezogen werden muss.

Wie in Tab. 4 dargestellt sollte bei einer LMNA, soweit wie möglich, auf jegliche sportliche Aktivität verzichtet werden.

**Table 4:** 

Risiko	LMNA-Typ	Freizeitaktivität	Schulturnen	Wettkampfsport	Bemerkungen
IR	Genetisch pos./phänotyp. neg.	+/−	+/−	−	Keine
HR	Genetisch pos./phänotyp. pos.	−	−	−	Evtl. ICD

*Beachte*: Sportempfehlungen sind immer Einzelfallentscheidungen nach detaillierter Aufklärung der Patienten

*ICD* implantierbarer Kardioverter-Defibrillator*, IR* „intermediate risk“, *HR* „higher risk“; *+* erlaubt, *–* nicht erlaubt

### Long-QT-Syndrom

Das LQTS ist eine vererbte Ionenkanalerkrankung, die durch eine verzögerte Repolarisation typischerweise polymorphe Torsaden-artige ventrikuläre Tachykardien auslösen kann. Der Verdacht auf ein LQTS ergibt sich, wenn in einem Ruhe-EKG oder in einem EKG 4 min nach maximaler Belastung mithilfe der Bazett-Formel eine QTc-Zeit > 470 ms für männliche bzw. > 480 ms für weibliche Probanden ermittelt wird. Eine QTc-Zeit > 500 ms gilt als beweisend [28].

Die Prävalenz ist in den letzten Jahrzehnten ständig gestiegen und beträgt zurzeit 1:2000 [17]. Bis dato sind zahlreiche LQTS-Mutationen bekannt, wobei LQTS1 (*KCNQ1*), LQTS2 (*KCNH2*) sowie LQTS3 (*SCN5A*) 75 % der genetischen Diagnosen stellen [29]. Die klinische Symptomatik reicht von Palpitationen über Synkopen (durch kurz anhaltende VT) bis zum SCD, wobei die zugrunde liegende Mutation, das Geschlecht und die QTc-Zeit entscheidenden Einfluss haben [30]. Bis zum 13. Lebensjahr haben Jungen mit einem LQTS1, danach Mädchen mit einem LQTS2 die höchsten Ereignisraten; die niedrigste Ereignisrate haben Kinder mit einer QTc-Zeit < 500 ms, wenn bisher keine Synkopen aufgetreten sind [31].

Bei jedem LQTS-Indexpatienten ist eine generationenübergreifende familiäre Abklärung einzuleiten

Da die Ausprägung der typischen EKG-Muster sehr variabel sein kann, ist die korrekte Diagnosestellung mitunter schwierig. Der Bekanntheitsgrad des LQTS jedoch und die zunehmende Zahl von EKG mit automatischer QTc-Zeit-Berechnung, die im Rahmen anderer Untersuchungen abgeleitet werden, lassen die Zahl der Indexpatienten in der Rhythmusambulanz stetig steigen. Die Zuweisung nach einem kardialen Ereignis ist in der heutigen Zeit eher selten. Bei jedem Indexpatienten muss eine generationenübergreifende familiäre Abklärung eingeleitet werden; der Anteil der Spontanmutationen beim LQTS beträgt etwa 10 % [32].

Therapeutisch ist beim LQTS1, weniger beim LQTS2, ist die nichtselektive β‑Blocker-Therapie (Propranolol) gut wirksam; Nadolol soll bei den 3 häufigsten Genotypen das Arrhythmierisiko im Vergleich ohne Therapie senken [33]. Beim LQTS3 können Na-Kanal-Blocker zu Einsatz kommen [34]. Bezüglich der Medikamentenauswahl muss kritisch angemerkt werden, dass Nadolol oder Mexiletin in vielen Ländern – so auch in Deutschland und Österreich – nicht regulär zur Verfügung stehen.

Bei jedem Beratungsgespräch sollte auf die genotypischen Trigger – für das LQTS1 sind dies körperliche Belastung (*cave*: Schwimmen), beim LQTS2 emotionale oder akustische Reize – als mögliche vermeidbare Auslöser hingewiesen werden. Dass auch Medikamente die QTc-Zeit signifikant verlängern können, wird allen Patienten eindringlich vermittelt, die Umsetzung in der Praxis scheitert jedoch manchmal und kann zu gefährdenden Situationen führen. Diverse Internetseiten helfen, den Überblick zu bewahren (z. B. https://www.sads.org).

Die unterschiedlichen klinischen Expressionen bei Patienten mit verschiedenen LQTS-Varianten erfordern auch eine differenzierte Beratung bezüglich körperlicher Aktivität, zumal zahlreiche Mutationsträger klinisch nie symptomatisch werden [35]. Ziel der individuellen Entscheidung, basierend auf den dokumentierten QTc-Zeiten und dem klinischen Verlauf, sollte sein, eine Teilnahme an Aktivitäten, soweit wie möglich, zu erlauben, ohne die Patienten auf der anderen Seite durch eine zu liberale Einstellung zu gefährden. Das diesbezügliche Vorgehen der Autoren ist in Tab. 5 zusammengefasst.

**Table 5:** 

Risiko	Typ	Freizeitaktivität	Schulturnen	Wettkampfsport	Bemerkungen
*LQTS 1*					
LR	LQTS 1, genetisch pos./phänotyp. neg.	+	+	+	Kein Wassersport
IR	LQTS 1, QTc-Zeit 460–500 ms	+	+/−	+/−	Kein Wassersport
IR	LQTS 1, QTc-Zeit > 500 ms	+	+/−/−	−	Kein Wassersport
HR	LQTS 1 + CE unter β‑Blocker-Therapie	−	−	−	Kein Wassersport Evtl. LCSD, ICD
HR	LQTS 1 zu Beginn der Therapie	−	−	−	Kein Wassersport Evaluationsphase (3 bis 6 Monate)
*LQTS 2*					
LR	LQTS 2, genetisch pos./phänotyp. neg.	+	+	+	Kein Schießsport, kein Biathlon
IR	LQTS 2, QTc-Zeit 460–500 ms	+	+/−	+/−	Kein Schießsport, kein Biathlon
IR	LQTS 2, QTc-Zeit > 500 ms	+	+/−/−	−	Kein Schießsport, kein Biathlon
HR	LQTS 2 + CE unter β‑Blocker-Therapie	−	−	−	Kein Schießsport, kein Biathlon Evtl. LCSD, ICD
HR	LQTS 2 zu Beginn der Therapie	−	−	−	Kein Schießsport, kein Biathlon Evaluationsphase (3 bis 6 Monate)
*LQTS 3*					
LR	LQTS 3, genetisch pos./phänotyp. neg.	+	+	+	Keine
IR	LQTS 3, QTc-Zeit 460–500 ms	+	+	+/−	Keine
IR	LQTS 3, QTc-Zeit > 500 ms	+	+/−/−	−	Keine
HR	LQTS 3 + CE unter β‑Blocker-Therapie	−	−	−	Evtl. LCSD, ICD
HR	LQTS 3 zu Beginn der Therapie	−	−	−	Evaluationsphase (3 bis 6 Monate)

*Beachte*: Sportempfehlungen sind immer Einzelfallentscheidungen nach detaillierter Aufklärung der Patienten

*CE* „cardiac events“ (rhythmogene Synkope, Torsade de pointes, Kammerflimmern), *genetisch positiv* legt den Genotyp fest, *HR* „higher risk“, *IR* „intermediate risk“, *LCSD* „left cardiac sympathetic denervation“, *LQTS 1–3* Long-QT-Syndrom, Typen 1–3, *LR* „low risk“, *phänotypisch negativ* heißt keine CE und normale QTc-Zeiten in allen verfügbaren EKG; *+* erlaubt, *–* nicht erlaubt

### Brugada-Syndrom

**Figure 4:**
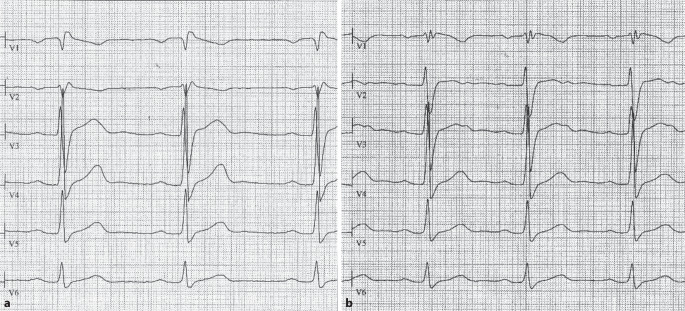

Das BrS ist durch spezifische ST-Strecken-Hebungen in den rechtspräkordialen Ableitungen charakterisiert; diese können durch die i.v.-Gabe von natriumblockierenden Antiarrhythmika (Ajmalin oder Flecainid) verstärkt werden. Die aus dem Erwachsenenbereich stammende Definition ist für Kinder und Jugendliche nur bedingt hilfreich, da die typischen sattelförmigen ST-Hebungen in dieser Altersgruppe kaum vorkommen und ein Ajamalin‑/Flecainidtest nur selten durchgeführt wird. Aus eigener Erfahrung ist die typische Präsentation im EKG das Bild eines „komischen“ oder atypischen Rechtsschenkelblocks (RSB; Abb. 4). Bei jedem Patienten mit einem „kompletten RSB“ sollte auch an die Möglichkeit eines BrS gedacht werden. Durch Variation der Interkostalräume zur Registrierung der rechtspräkordialen Ableitungen (V_1_ und V_2_ um einen bzw. 2 Interkostalräume höher platziert als normal) kann diese Veränderung evtl. leichter detektiert werden.

Die generationenübergreifende Aufarbeitung identifiziert BrS-gefährdete Kinder oder Jugendliche

Sehr selten sind Kinder oder Jugendliche die Indexpatienten in der Familie, viel häufiger kommen diese aus dem Erwachsenenbereich. Die generationenübergreifende Aufarbeitung führt dann auch zu vermeintlich betroffenen Kindern oder Jugendlichen. Allerdings ist die Interpretation der genetische Analyse beim BrS schwierig, da bisher zumindest 20 erkrankungsbezogene Gene bekannt sind; diese werden nicht wie beim LQTS streng nach den Mendel-Gesetzen vererbt [36]. Die typischen *SCN5A*-Mutationen finden sich nur bei etwa 21 % der phänotypischen BrS-Patienten [37], die Patienten weisen dann jedoch signifikant mehr epikardiale elektrophysiologische Abnormitäten im RVOT auf und zeigen auch einen klinisch maligneren Verlauf [38].

Das BrS (und andere GAS) sind für 10–20 % der SCD im Säuglingsalter verantwortlich [39]. Beide Tatsachen zusammen – nämlich die möglicherweise nichtverwertbare genetische Untersuchung und das Risiko eines SCD bereits im Säuglingsalter – erschweren das klinische Management. Das Risiko des SCD durch Kammerflimmern wird typischerweise durch Fieber provoziert. Die Autoren empfehlen daher bei allen Säuglingen von BrS-Indexpatienten – unabhängig, ob genetisch positiv oder negativ – im 1. Lebensjahr die stationäre Aufnahme bei Fieber, Durchfall oder Erbrechen, einerseits zur aggressiven Fiebersenkung, andererseits zur Dokumentation eines evtl. induzierten BrS-typischen EKG. Im Rahmen der durch die „coronavirus disease 2019“ (COVID-19) verursachten Pandemie zählen BrS-Patienten – v. a. mit einem spontanen Typ-1-EKG, nach Synkopen oder ICD-Implantation – zur Risikogruppe bei Impfwunsch. Diesen Patienten wird eine prophylaktische antipyretische Therapie, zu Hause oder evtl. unter stationären Bedingungen empfohlen [40].

Ein medikamentöser Ansatz ist Chinidin, bei phänotypisch positiven, symptomatischen Patienten ist die einzige effektive Maßnahme zur Prävention des SCD die Implantation eines ICD. Bei symptomatischen Erwachsenen stellt die v. a. epimyokardiale Ablation einen erfolgreichen Therapieansatz dar [41].

Sportliche Aktivität ist bei BrS-Patienten kein direkter Trigger und prinzipiell erlaubt, es sollten jedoch indirekte Trigger, wie hohe Körpertemperatur oder Elektrolytveränderungen durch starken Flüssigkeitsverlust, vermieden werden (Tab. 6).

**Table 6:** 

Risiko	BrS-Typ	Freizeitaktivität	Schulturnen	Wettkampfsport	Bemerkungen
LR/IR	Genetisch pos./phänotyp. neg.	+	+	+	Keine
HR	Genetisch pos./phänotyp. pos.	−	−	−	Evtl. ICD

*Beachte*: Sportempfehlungen sind immer Einzelfallentscheidungen nach detaillierter Aufklärung der Patienten

*HR* „higher risk“, *IR* „intermediate risk“, *LR* „low risk“, *phänotypisch positiv* sind Patienten mit spontanem oder medikamentös induziertem Typ-1-EKG oder nach einer rhythmogenen Synkope; *+* erlaubt, *–* nicht erlaubt

### Katecholaminerge polymorphe ventrikuläre Tachykardie

Die CPVT manifestiert sich in emotional oder durch körperliche Aktivität getriggerten polymorphen ventrikulären Extrasystolen, die typischerweise in Korrelation mit der ansteigenden Herzfrequenz zunehmen und in bidirektionale ventrikuläre Tachykardien mit oder ohne Synkopen oder in Kammerflimmern mit dem Risiko des SCD übergehen können. Die geschätzte Prävalenz beträgt 1:10.000 [42], Erkrankungsfälle treten jedoch schon im Kindes- und Jugendalter auf [43], wobei die CPVT die am häufigsten tödlich verlaufende angeborene Ionenkanalerkrankung ist [44]. Die beiden häufigsten Mutationen finden sich im kardialen Ryanodinrezeptorkanal (*RYR2*; autosomal-dominant vererbt) und im Calsequestrin (*CASQ2*; autosomal-rezessiv vererbt, heterozygote Mutationsträger sind normalerweise gesund) [45]. Die molekulargenetische Diagnostik sichert die Diagnose und erlaubt die weitere gezielte Abklärung im familiären Umfeld.

Die CPVT ist die am häufigsten tödlich verlaufende angeborene Ionenkanalerkrankung

Das Ruhe-EKG ist unauffällig; eine häufige Zuweisungsdiagnose sind VES. Bei Dokumentation von VES vom „uncommon type“ (diese sistieren nicht bei Belastung) muss immer an eine CPVT gedacht werden. Das Belastungs-EKG kann mit dem Auftreten von polymorphen ventrikulären Extrasystolen die Diagnose erhärten.

Als medikamentöse Therapie wird eine Kombination aus β‑Blockern (Klasse I), Flecainid (Klasse IIa) und Ivabradin verabreicht, die Medikamentenwirkung ist jedoch individuell extrem unterschiedlich und zumeist besser bei *RYR2*-Mutationen [46]. Die linksseitige kardiale Sympathektomie (Klasse IIB) ist bei unzureichender medikamentöser Wirkung indiziert, die ICD-Implantation ist bei therapieresistenten ventrikulären Tachykardie nötig, jedoch nicht unproblematisch, da zahlreiche Komplikationen, auch ein „elektrischer Sturm“ induziert werden können [46]. Die ICD-Implantation sollte so lange wie möglich vermieden werden.

Aufgrund des malignen Verlaufs muss bei phänotypisch positiver CPVT auf jede sportliche Aktivität verzichtet werden, aber auch im Alltag sollten durch Lifestyle-Modifikation entsprechende Trigger (z. B. dem Bus nachlaufen …) vermieden werden (Tab. 7). *Beachte*: Dies kann in der Praxis schwierig sein, muss jedoch konsequent umgesetzt werden. Hilfreich könnte das Verwenden von Pulsuhren sein, die beim Erreichen der Cut-off-Herzfrequenz – es ist dies die Herzfrequenz, bei der in der Ergometrie oder im Holter-EKG die ersten VES auftreten – alarmieren.

**Table 7:** 

Risiko	CPVT-Typ	Freizeitaktivität	Schulturnen	Wettkampfsport	Bemerkungen
HR	Genetisch pos./phänotyp. neg.	+/−	+/−	+/−	Keine
HR	Genetisch pos./phänotyp. pos.	−	−	−	Evtl. ICD

*HR* „high risk“, *phänotypisch positiv* polymorphe ventrikuläre Tachykardien unter Belastung, *+* erlaubt, *–* nicht erlaubt

## Fazit für die Praxis

- Die Therapie von tachykarden Rhythmusstörungen in der Pädiatrie erfordert immer deutlicher einen generationenübergreifenden Ansatz.
- Dieser beginnt bei fetalen Tachykardien mit der professionellen Betreuung durch die Geburtshelfer, setzt sich bis zur Behandlung durch Erwachsenenkardiologen fort und schließt die intensive Zusammenarbeit auf dem technisch hoch anspruchsvollen Gebiet der elektrophysiologischen Untersuchungen ein.
- Besonders wichtig ist der generationenübergreifende Ansatz bei der Betreuung von Familien mit genetischen Arrhythmiesyndromen. Die intensive Kooperation mit Genetikern, Allgemeinmedizinern und Erwachsenenkardiologen kann dazu beitragen, gefährdete Patienten frühzeitig zu erkennen und einen plötzlichen Herztod mithilfe prophylaktischer Maßnahmen zu verhindern.
- Möglicherweise ist dieser Ansatz der generationenübergreifenden Familienbetreuung auch effektiver als aufwendige und teils problematische Screeninguntersuchungen.
